# Preparing national tiered laboratory systems and networks to advance diagnostics in Africa and meet the continent’s health agenda: Insights into priority areas for improvement

**DOI:** 10.4102/ajlm.v9i2.1103

**Published:** 2020-09-21

**Authors:** Pascale Ondoa, Nqobile Ndlovu, Mah-Sere Keita, Marguerite Massinga-Loembe, Yenew Kebede, Collins Odhiambo, Teferi Mekonen, Aytenew Ashenafi, Amha Kebede, John Nkengasong

**Affiliations:** 1 African Society for Laboratory Medicine, Addis Ababa, Ethiopia; 2 Amsterdam Institute for Global Health and Development, Academic Medical Centre, Department of Global Health, University of Amsterdam, Amsterdam, Netherlands; 3 Africa Centres for Disease, Control and Prevention, Addis Ababa, Ethiopia

## Background

Access to diagnostics remains sub-optimal in Africa due to limited human, financial and technical resources that affect various components of the health system.^1,2^ Additionally, the lack of standardised systems for evaluation and registration of diagnostics^3^ cripples the introduction of better technologies, representing missed opportunities to address healthcare challenges. Examples of poor access to diagnostics and its dramatic consequences are numerous. Despite strong vertical control programmes, 40% of HIV-infected patients on antiretroviral therapy do not receive the recommended yearly HIV viral load monitoring test.^4^ In 2016, a dramatic 21% of children born to HIV-positive mothers were reported as not receiving early infant diagnosis testing before age 8 weeks in West and Central Africa.^5^ Data from the World Health Organization (WHO) global tuberculosis database of 2016^6^ also indicate that drug-resistant tuberculosis is largely missed in Africa. In addition to 70% of patients not being notified, a rifampicin susceptibility test was available to less than 10% of patients in 23 of 47 countries and second-line resistance testing was available only in 60% of the countries on the continent.^7^ The picture is equally worrisome for diseases that are poorly or not supported through dedicated programmes. Nine in 10 individuals carrying the hepatitis B or C virus have never been tested, while these infections are estimated to cause 60% of liver cancers and an epidemic larger than that caused by HIV.^8^ In a study conducted in Senegal in 2015–2016, less than 30% of pregnant women attending antenatal care at the primary healthcare level had access to the minimum panel of screening tests for the most common clinical conditions threatening maternal and child health.^9^ Almost half of the mortality cases associated with cervical cancer are due to late detection of the disease.^10^ More recently, it appeared that when the first coronavirus disease 2019 (COVID-19) case was reported in Egypt in February 2020, only 2 of the 55 countries on the continent were capable of detecting the severe acute respiratory syndrome coronavirus 2 (SARS-CoV-2). Insufficient access to in vitro diagnostics (IVD) at all levels of healthcare delivery in low-income and middle-income countries reduces access to life-saving treatments, impairs the delivery of quality healthcare and compromises progress towards Universal Health Coverage in Africa. Implementation of simplified, more robust and more affordable diagnostic options has been proposed to increase access to diagnostics in low-resource settings. Rapid diagnostic tests that can be performed at the community level or through self-testing have transformed the management and prevention of several diseases such as HIV infection, malaria and diabetes.^11,12,13^ Point-of-care molecular technologies, such as the GeneXpert® or the m-Pima®, have been demonstrated to increase specificity and simplicity of testing, while reducing the turnaround time for test results^14,15^ for improved patient retention and management. Despite addressing critical testing processes or delivery gaps, innovative or conventional diagnostic technologies frequently fail to translate into tangible public health outcomes.^16,17,18^

The failure of diagnostics to reach a large proportion of the population in need of it can partly be linked to implementation approaches that are designed for the site level, with oversight of the tiered laboratory network requirements and insufficient attention given to the underlying laboratory systems (e.g. supply chain, workforce, finance, etc.). Complementing the Maputo Declaration of 2008,^19^ the Freetown Declaration of 2015^20^ underscores that delivering diagnostic services in the context of functional, integrated national laboratory networks is the recommended strategy for providing maximum population coverage and cost-effective utilisation and delivery of diagnostic services in resource-limited settings.

Apart from selected disease-dedicated investments (e.g. HIV, tuberculosis), national tiered laboratory networks generally remain grossly underfunded with mild to critical dysfunctions in the underlying laboratory systems. In addition to falling short in the provision of optimal access to essential clinical diagnostics, the current sub-optimal capacity of tiered laboratory networks translates into weaknesses in the detection component of the ‘prevent, detect, respond’ framework of health security^21,22^ Missed opportunities to stop onward transmission of infectious diseases and to detect and prevent non-communicable diseases hinder efforts to reduce the burden of illnesses in Africa. The WHO estimates that the resulting loss of annual productivity due to the heavy disease burden in Africa equalled $2.4 trillion and 630 million years of healthy lives in 2015.^23^

Despite the call for more attention in building effective public health laboratory systems,^24^ which was made in the aftermath of the Ebola epidemic of 2015, few data exist that allow quantification of the overall performance of national laboratory networks and systems across diseases. This lack of information prevents the design of impactful interventions and hinders the uptake of the otherwise large and relevant range of diagnostic technologies available.

In this report, the African Society for Laboratory Medicine (ASLM) and the Africa Centres for Disease Control and Prevention (Africa CDC) share a unique insight into some of the most critical areas for improvement to bridge the gap between the capacity of laboratory networks and the promises of diagnostic technologies.

## Observations

### Root causes of dysfunction in laboratory networks are not sufficiently measured or addressed

**FIGURE 1:**
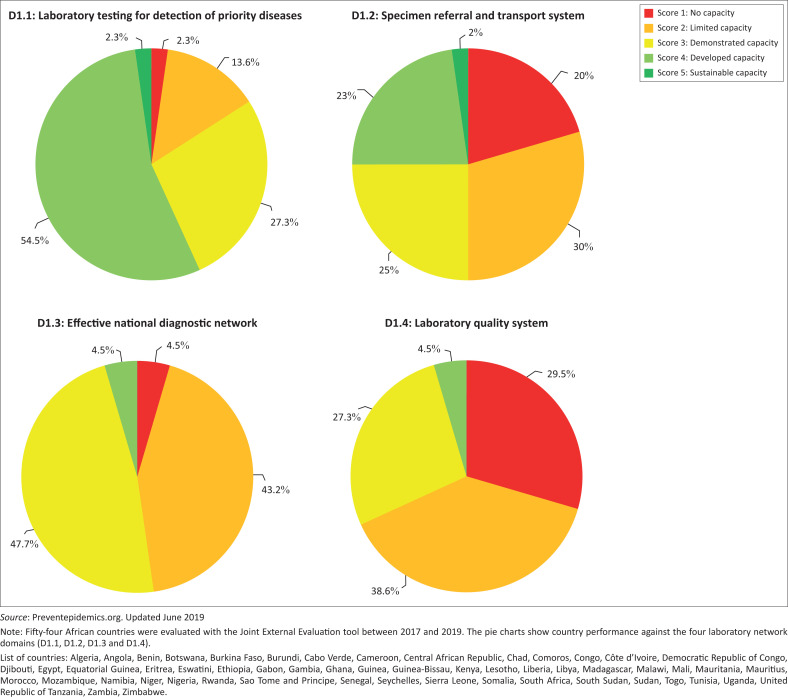
Proportion of African countries (*N* = 54) at various levels of capacity in the area ‘laboratory network’ of the Joint External Evaluation tool.

**FIGURE 2:**
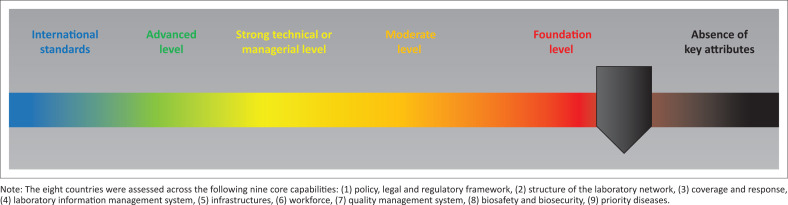
Overall level of laboratory network functionality in eight countries assessed.

Actionable data on integrated laboratory network functionality are scarce. The WHO Joint External Evaluation^25^ tool provides a high-level overview of the performance of the national laboratory network across four domains: (D1.1) laboratory testing for the detection of priority diseases, (D1.2) specimen referral and transport system, (D1.3) effective national diagnostic network and (D1.4) Laboratory Quality System, as part of the evaluation of entire national health systems to support compliance with International Health Regulations requirements. Data from 54 countries^21^ indicate that quality management and sample referral systems are the most neglected areas across the continent with 68.1% and 50.0% of countries having none to only a basic capacity in place (Figure 1), further aggravating the gaps in diagnostic networks with issues related to coverage and reliability of testing. More than 48.0% of countries have gaps in defining or providing access to tier-specific laboratory testing strategies, while more than 84.0% of the countries assessed demonstrated sustainable capacity for laboratory testing to support the surveillance of 10 priority diseases. The Global Health Security Agenda laboratory network (LABNET^26^) scorecard was developed to complement the Joint External Evaluation tool and to provide more granularity on nine core capabilities of the laboratory network and systems, enabling the identification of specific gaps and related root causes. In eight countries where this evaluation was conducted, the average performance of laboratory networks ranged from none to basic capacity across the nine core capabilities assessed (Figure 2), highlighting the presence of many common disabling factors (Table 1), including:

- **Weak laboratory governance**. In several countries, no directorate of laboratories exists or it is not directly placed under the authority of the Ministry of Health, preventing adequate coordination of laboratory services and limiting the sphere of operation of the laboratory vis-à-vis the other health sectors. Unclear assignment of administrative versus technical tasks and mandates between directorates and national public health institutes and national reference laboratories also prevents the overall development and enforcement of regulations related to various aspects of diagnostics (such as IVD evaluation and registration, definition and updates of tier-specific minimal testing packages, laboratory staffing norms or definition and implementation of quality standards).

**TABLE 1:** Most critical gaps in laboratory network core capabilities were identified through the laboratory network assessment of eight sub-Saharan African countries between 2017 and 2019, and their implications for diagnostic services.

Core capabilities assessed	Most critical common weaknesses (score 0–1)	Implication on diagnostic services
Policy, legal and regulatory framework	The nine essential public health functions† are not legally enforceable and are not incorporated in national laboratory strategic plans No national system for licensing laboratories No dedicated budget for laboratory services for both routine and emergency situations	Inconsistent compliance of diagnostic services to the principles of biosafety, biosecurity, waste management, and the protection of the environment. Inconsistent application of diagnostic services to outbreak response, food safety and disease notification Facilities with low capacities are allowed to deliver diagnostic services Diagnostic services cannot be optimally organised and coordinated, also during situations of disease outbreaks
Structure of the tiered network	The network does not incorporate testing activities at community level	Results of rapid and point-of-care testing at community level are not supervised and quality controlled
Network coverage and rapid response	Lack of updated data on the geographic information system location of laboratory capacity Minimal testing packages are not defined at all tiers Diagnostic services are not accessible at points of entry No national plan to mobilise laboratories in case of health emergencies	Implementation of diagnostics unlikely to cover optimal testing needs and to ensure cost-effectiveness of services Diagnostics are not used at the most relevant levels, reducing the public health impact of diagnostics strategies Diagnostics cannot contribute to control the spread of diseases across borders Diagnostics cannot optimally contribute to the response to health emergencies
Laboratory information management system	No standardised test request and result return forms at national level No central unit for health data analysis No systems to ensure confidentiality and anonymity of test results	Test demand and result utilisation are compromised even when diagnostics are available Diagnostics cannot optimally inform public health interventions Clients do not trust the diagnostic services, which undermines the demand for testing
Infrastructure, reagents and supplies	Building norms not applied for the construction of testing facilities Testing facilities are not maintained, with frequent interruption of water, electricity and Internet supply Insufficient system to forecast and adequately supply reagent in routine and emergency situations	Diagnostic testing is done in unsafe, unsecured or illegal locations Diagnostics in place are not used according to biosafety and biosecurity requirements. Instruments cannot be operated Diagnostic services are interrupted, even in situations of emergency
Workforce	Insufficient training for laboratory management Incomplete, inadequate or no staffing plan Overall shortage of staff to deliver testing services No human resources strategy specifically and comprehensively addressing the laboratory workforce	Overall diagnostic services are ineffective at both facility and national level The correct execution of diagnostics is compromised
Quality management systems	Inconsistent internal quality control procedures Quality focal point not in place in all laboratories No national norms for laboratory certification and accreditation	Unreliable test results Quality of test results cannot be verified
Biosecurity	Insufficient availability of biosecurity of adequate level No systems to store and archive samples, including dangerous pathogens Gaps in waste management systems	Unreliable test results (e.g. AST) Unsafe diagnostic testing for staff and environment Missed opportunities for in-house or external validation of diagnostics
Priority diseases	Poor capacity for isolating AMR bacteria Insufficient reporting of AMR	AMR testing does not feed into representative AMR surveillance systems List of pathogens and antibiotics of importance are not updated and many irrelevant AST are conducted

AMR, antimicrobial resistance; AST, antibiotic susceptibility test.

† , Essential public health functions are: Disease prevention, control and surveillance; Integrated data management; Reference and specialized testing; Environmental health and protection; Food safety; Laboratory improvement and regulation; Policy development; Public health preparedness and response; Public health related research; Training and Education; Partnership and communication. The NRL or NPHL does not have to perform them all but must ensure that they are in place.

Two countries with strong laboratory governance include Ethiopia, through the Ethiopian Public Health Institute, and Uganda, through the Directorate of Laboratory Services of Uganda. They offer good examples of best practices in the management of the laboratory systems and networks. These countries report updated national policies and strategic plans, budget lines earmarked for laboratory functions and regulations defining and enforcing laboratory clinical and public health functions from the reference level to the community level:

- **Missed opportunities to prioritise diseases for both surveillance and care**. Countries do not have access to up-to-date and fit-for-purpose epidemiological data to prioritise diseases of public health importance using a risk-based approach. Often, the WHO list of 43 pathogens for surveillance serves as a *de facto* national list of ‘priority’ pathogens. The WHO recommends prioritising 10 pathogens^27^ to ensure that communicable diseases with the most severe morbidity and mortality are effectively screened, confirmed and the treatment monitored at the various tiers of the diagnostic network. Countries implementing the Global Health Security Agenda prioritise 10 core tests covering International Health Regulations immediately notifiable diseases among the WHO top 10 causes of death in low-income and middle-income countries. The Integrated Disease Surveillance and Response framework of the WHO regional office for Africa recommends prioritising epidemic-prone diseases targeted for eradication and elimination, and endemic diseases, resulting in a list of 19 diseases. In practice, these overlapping recommendations are difficult to interpret. The plethora of ‘prioritised’ diseases complicates the implementation of robust tier-specific packages and surveillance systems that can support both epidemiology reporting and clinical care.
- **Insufficient attention to evidence-based management of laboratory networks**. None of the countries assessed had well-defined processes to routinely monitor the performance of the laboratory network without the support of external partners. This situation precludes the establishment of a quality assurance loop of laboratory networks and systems. Ultimately, the lack of monitoring and evaluation systems (i.e. lack of key performance indicators for the laboratory sector and of responsible units to collect, analyse and act upon the data) for laboratory networks undermines the return on investment of most capacity strengthening interventions aimed at advancing diagnostic services.

#### Recommendations

Strengthening of integrated quality management and specimen referral systems are the most urgent interventions needed, the outcomes of which should be evaluated against the advancement of Joint External Evaluation scores. Various initiatives funded by global stakeholders are ongoing (e.g. United States Agency for International Development, United States President’s Emergency Plan for AIDS Relief, Global Fund) with the potential to be transitioned to and sustained by stronger and empowered national laboratory leadership with the political support of Africa CDC. The ASLM and their partners can work together at formulating clearer guidance on the respective mandates of directorates of laboratories and national public health institutes (including the monitoring and evaluation framework for laboratory network functions) as well as advocating to empower the laboratory sector. These efforts align with the recommendations of the Maputo and the Freetown Declarations. Assisting countries to define tier-specific testing packages that address the needs of clinical diagnostics and disease surveillance is another important intervention with the potential to guide the introduction of diagnostics at the levels where they are most needed and cost-effective. We recommend that every country conduct an external or self-evaluation of their laboratory systems once a year or once every two years as part of a continuous quality improvement cycle for their national tiered laboratory network. While the Joint External Evaluation tool provides high-level dashboards for a set of four indicators, the LABNET scorecard is designed to guide countries in selecting specific and feasible interventions most likely to tackle the root causes of the identified dysfunctions across a comprehensive set of indicators.

### Laboratory networks are not configured to support diagnostic services that are integrated, cost-effective and with maximum population coverage

Mutualising scarce resources for most cost-effective health services is (or should be) a constant concern in low-resource settings. A data-driven configuration of a national laboratory network can support the design of faster and more affordable sample transportation routes towards testing hubs. From the specimen referral landscape, assessments that were performed during 2015 and 2016 in eight countries by ASLM under the Global Health Security Agenda laboratory strengthening effort revealed that a certain level of integration of the specimen transport system (STS) can exist, often between HIV and tuberculosis programmes. The STSs are generally fragmented, working in parallel, using different transport mechanisms depending on the disease programme, are funded by various donors and have challenges in terms of cost-effectiveness, turnaround time and coverage. A few countries, such as Ethiopia, Rwanda and Uganda, have highly integrated specimen referral networks that cater to any disease programme and span across diagnosis to surveillance, detection and response. Ideally, this disease-agnostic approach should be developed such that individual STSs are effectively and efficiently networked, and that any type of specimen can be easily and seamlessly moved from where it originates to the appropriate diagnostic equipment.

Addressing the common problem of fragmented and disease-specific STSs, ASLM coordinated the development of a standardised STS assessment and development toolkit,^28^ addressing multiple diseases, surveillance and clinical diagnostic needs, and factoring in different modes of transportation. One country, Burkina Faso, was able to use the findings of the assessment to establish a new specimen referral system for surveillance that is now being built upon, scaled up and integrated to cover any disease programme.^29^

**FIGURE 3:**
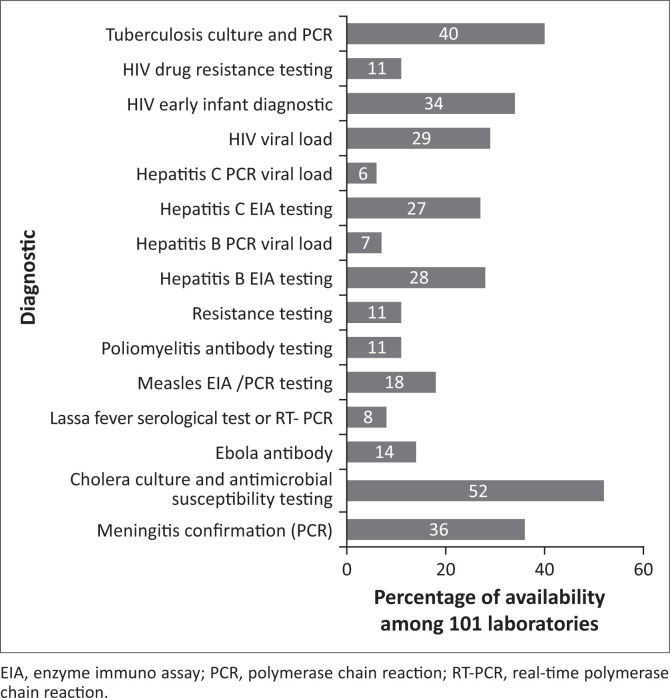
Gaps between actual diagnostic capacity and World Health Organization recommendations for minimal testing package in 101 level 4 and level 3 laboratories in 12 African countries (data from 2018 to 2019).

The availability of up-to-date geo-localised information about laboratory network capacity provides evidence on which to base the process of defining optimal service configurations, including the shortest routes for sample transportation, maximum population coverage for testing services, cost-effective supply chains, or opportunities for testing integration. Initiatives aimed at collecting geographical information system data on laboratory capacity are gaining momentum to improve the performance of HIV and tuberculosis control programmes.^30,31^ The ASLM and Africa CDC have implemented a continent-wide laboratory capacity mapping programme (LabMap^32^) across diseases, based on open-source tools (Ona [Ona Systems Inc., Nairobi, Kenya], PlanWise [InSTEDD, Sunnyvale, California, United States]^33^) and fostering country ownership. This system allows the easy collection, curation and analysis of geospatial data to make informed decisions on national laboratory networks and is interoperable with the District Health Information System (DHIS2, University of Oslo, Norway) 2^34^. Thirteen of the 54 countries on the continent (24%) are currently using the system under the coordination of Africa CDC’s regional coordinating centres.^35^ Among the 101 level 4 and level 3 laboratories assessed, only 40% had the capacity to conduct culture or polymerase chain reaction-based tuberculosis diagnosis, 11% and 34% could perform HIV drug resistance genotyping and early infant diagnosis, and 36% could run confirmatory testing for meningitis through culture or polymerase chain reaction (Figure 3). Using LabMap data from 2018, we determined that in Niger,^36^ eight facilities at level 3 are conducting meningitis testing, including serology, bacterial culture and nucleic acid testing, covering a total of 7.9 million people (40% of the Nigerien population) within a 2-hour drive radius. Only one facility (covering 2.8 million people, 14% of the population) is equipped to conduct polymerase chain reaction for the differential diagnosis of pathogens causing meningitis, which is critical for the swift adjustment of antibiotic therapy. PlanWise calculated that three strategically located level 3 laboratories involved in meningitis testing could be upgraded with a maximum impact reaching out to an additional 4.1 million (20%) inhabitants. PlanWise and other similar software like the Supply Chain Guru from Llamasoft^37^ offer the opportunity to calculate the best options to improve the laboratory network testing capacity and coverage, including upgrading existing facilities, building new laboratories or configuring specimen referral and supply chain routes.

#### Recommendations

Data-driven optimisation of laboratory networks is an important management activity that can support essential public health and clinical functions. Key considerations of any optimisation exercise should include at least: the structure of the tiered laboratory networks, (essential) testing needs for both clinical diagnosis and disease surveillance, and opportunities for test integration. Specimen referrals are important systems underlying national laboratory networks. Taking a network approach for developing integrated STSs may provide greater cost savings, efficiencies, increased coverage and increased access. This approach can begin by mapping and optimising the overall referral network based on the existing diagnostics network (or any upcoming changes), reducing redundancies where possible and leveraging existing or new systems to create economies of scale, clearly planning and budgeting for the optimised network and implementing the new approach. Any potential benefits should be clearly estimated and used to convince governments of the approach and gain buy-in from other stakeholders as well.

Regular collection and updating of (geo-located) laboratory capacity information is necessary to inform network optimisation exercises. Such activities could be enforced as part of laboratory registration or licensing and re-licensing processes under the coordination of the directorate of laboratories.

### Most diagnostic services delivered through laboratory networks are not quality assured

Unreliable diagnostic tests can compromise the quality of healthcare delivery. A laboratory quality management system (LQMS) is a formalised system that documents processes, procedures and responsibilities for achieving an international standard of quality. The implementation of LQMS was identified as a priority to strengthen laboratory services in Africa^38,39^ a decade ago. As part of this momentum effect, various tools and frameworks have been developed and disseminated to guide laboratories towards LQMS implementation and International Organization for Standardization 15189 accreditation. Some of these resources have a regional or a disease-specific focus; these include the Strengthening Laboratory Management Toward Accreditation, the Stepwise Laboratory Quality Improvement Process Towards Accreditation (SLIPTA), which also includes a tuberculosis-specific checklist, the LQMS training tool kit and the Laboratory Quality Services International group. To date, around 520 laboratories have received international accreditation on the continent. More than 370 of these accredited facilities are in South Africa and most of them belong to the public sector, highlighting geographic differences and the lagging behind of private laboratories. The SLIPTA is a WHO regional office for Africa programme assessing laboratory progress towards the International Organization for Standardization 15189 standards and with ASLM serving as the secretariat. Since its launch in 2012, around 430 laboratories implementing LQMS have been assessed across 17 countries (Table 2), illustrating the increasing awareness of national stakeholders with regard to quality requirements, as well as the commitment of the laboratory community to advance diagnostic services. However, this number is below the original target of 2500 enrolled laboratories set at the programme onset^40^ and represents a modest outcome at both the continental and country scale. A couple of countries have demonstrated impressive coverage of the SLIPTA programme (e.g. Botswana and Namibia, Table 2). However, assuming that only 50% of the total number of government laboratories are in hospitals (the target facilities to implement the International Organization for Standardization 15189 norm), the current number of laboratories engaged in SLIPTA represent only 0.3% of the total number of government laboratories in Nigeria and 3% in Kenya. Some bottlenecks with LQMS implementation using SLIPTA are inherent to the programme itself. The handling of certification requests and processes through countries’ Ministry of Health prioritises government over private laboratories. The lack of core funding supporting the programme translates into insufficient capacity for ASLM to cover the needs of an entire continent, not only to conduct audits but also to mentor laboratories towards quality improvement and accreditation. Benefitting from more stable United States President’s Emergency Plan for AIDS Relief funding, and despite reaching an impressive 1333 laboratories across various continents,^41^ the mentorship-focused Strengthening Laboratory Management Toward Accreditation programme is equally unable to comprehensively cover the needs of entire national laboratory networks. Advocacy efforts towards policymakers are also impaired by the lack of simple indicators linking LQMS implementation with improved health outcomes. Country-specific roadblocks have also been identified such as low political commitment and the lack of regulatory and policy frameworks guiding the expansion of LQMS as national programmes, adapted for each tier of laboratory networks. Recently, Continuous Quality Improvement initiatives targeting disease-specific testing (such as HIV) have been implemented. Although Continuous Quality Improvement directly addresses the quality of diagnostic testing through a problem-solving approach linked to patient outcomes, it still needs to be embedded into larger quality management programmes in order to contribute to sustainable outcomes supporting Universal Healthcare Coverage and International Health Regulations.

**TABLE 2:** Stepwise Laboratory Quality Improvement Process Towards Accreditation coverage of facilities in the public and private sector in 17 African countries (as of August 2019).

Country	Estimated number of laboratories in the public sector	Estimated number of laboratories in the private sector	Number of laboratories engaged in SLIPTA in the public and private sectors
Botswana	53	NA	23
Burkina Faso	107	112	3
Burundi	624	489	15
Cameroon	3279	NA	16
Côte d’Ivoire	420	113	11
Ethiopia	3949	1797	34
Ghana	2280	NA	21
Kenya	2245	NA	37
Malawi	1026	NA	19
Namibia	44	40	15
Niger	335	73	1
Nigeria	34423	NA	50
South Africa	221	NA	31
Tanzania	6213	NA	53
Uganda	1625	NA	60
Zambia	1608	NA	4
Zimbabwe	1630	NA	35

SLIPTA, Stepwise Laboratory Quality Improvement Process Towards Accreditation; NA, not available.

In addition to the poor coverage of LQMS implementation, most countries do not have systems in place to ensure that all testing sites comply with the basic components of quality assurance such as external quality assessments, proficiency testing schemes^42^ and various quality checks linked to post-market surveillance of IVD. Collectively, these observations suggest that dramatically high proportions of diagnostic tests delivered in African countries are not adequately quality assured. This inevitably results in laboratory test results that are incorrect or fraudulent, causing a lack of trust among clinicians and communities.

#### Recommendations

To overcome these challenges, the ASLM and WHO regional office for Africa launched SLIPTA version 2.0,^43^ which proposes to institutionalise SLIPTA at the country level, harness partner resources for SLIPTA funding and redefine the role of ASLM as a coordinating rather than an implementing body.

This strategic approach is currently being implemented through regional collaborations where the West African Health Organization and East, Central and Southern Africa Health Community are providing leadership to advance LQMS using the SLIPTA programme in West, Central East and Southern Africa, with ASLM serving as a high-level coordinator. According to this model, ASLM and its partners work at generating a sufficient pool of local SLIPTA auditors and laboratory mentors who can be mobilised to advance LQMS and conduct SLIPTA audits upon regional or national request. The efforts of Africa CDC to establish The Regional Integrated Laboratory Network (RISLNET) is also contributing to the extension of SLIPTA version 2.0, through the implementation of LQMS in national public health institutes, with subsequent deployment of the system in lower tiers of the national laboratory networks and across the private sector.

A couple of African countries like Ethiopia have stepped forward to ‘franchise’ the SLIPTA model as national programmes aiming to cover all laboratory facilities from the public and the private sector. The foundation of a country-led SLIPTA programme is the definition of national quality standards for diagnostic services at each level of service delivery, clearly describing which laboratory has the vocation to be accredited or certified and against which set of minimum quality standards.

### Critical shortage of pathologists compromises present and future benefits of laboratory diagnostics

Clinical pathologists are physicians trained in various disciplines of laboratory medicine, such as haematology, medical microbiology, transfusion medicine, clinical biochemistry or cytopathology.^44^ They provide highly specialised knowledge and leadership, ensuring the quality of the pre-analytical, analytical and post-analytical phases of testing, as well as critical information on the severity and prognosis of diseases based on test results. Clinical pathologists make sure that patients are started and maintained on the correct treatment regimen. In the United States and United Kingdom, the pathology workforce varies between 3 and 5 per 100 000 inhabitants.^45^ A quick desk survey conducted by ASLM reveals critical gaps in the clinical pathology profession in 10 countries of sub-Saharan Africa. Firstly, the mere definition of this profession is often not well understood, with most survey respondents only aware of anatomic pathologists but not clinical pathologists. Data from the College of Pathologists in East, Central and Southern Central Africa show a bias towards anatomical pathology (60%) compared to clinical pathology (18%) of the 119 registered pathologists (Dr Shahin Sayed, personal communication). Some countries also use pharmacists as ‘pathologists’,^46^ although this profession requires a background in medical studies, suggesting insufficient clinical interpretation of laboratory test results that might compromise patient outcomes. Secondly, an up-to-date inventory of pathologists by national professional councils seems to be lacking in many countries, suggesting that these highly specialised professionals are not adequately registered or certified in their respective countries. Thirdly, the ratio of pathologists (regardless of their specialty) is 5–350 times lower than ratios observed in the United States (Table 3), translating into gaps of more than 4000 pathologists for a country like Ethiopia or more than 6000 for a country like Nigeria. In most countries sampled, the number of pathologists is lower than the number of tier 2 and tier 3 hospitals (where pathology services are required), suggesting that clinical pathologists would have to serve in more than one facility to reduce the gaps. For example, 61 pathologists in Ethiopia have to support a total of 400 hospitals. The number of medical microbiologists (a sub-specialty of clinical pathology), at 0.5, is even lower in all countries sampled. This worrisome situation raises concerns about the sustainability and impact of current global and national efforts to establish diagnostic capacity for the control of antibiotic resistance on the African continent. The introduction of novel diagnostic technologies such as point-of-care testing at the community level or next generation sequencing at the reference level will only increase the need for specialised laboratory medicine professionals who are able to ensure the correct use and interpretation of diagnostic tests for improved patient and public health outcomes. Collectively, these data highlight severe gaps in general and clinical pathology, in particular in sub-Saharan African countries.

**TABLE 3:** Overview of numbers, coverage and needs of pathologists in nine surveyed countries of Africa (data from August 2019).

Countries surveyed	Total number of pathologists	Number of pathologists per 100 000 population	Times lower than United States ratio†	Number of pathologists needed‡
Ethiopia	61	0.05	70	4209
Sierra Leone	7	0.01	350	2443
Eswatini	1	0.09	39	38
Zambia	13	0.07	50	637
Rwanda	51	0.41	9	384
Nigeria	350	0.18	19	6456
Burkina Faso	42	0.21	17	658
Gabon	15	0.71	5	59
Kenya	131	0.25	14	1703
South Africa	216	0.37	9	1827

† , 3.5/100 000.

‡ , Using United States ratio as a reference.

#### Recommendations

In-country capacity for pathology training is commonly reported, with efforts by the College of Pathologists in East, Central and Southern Central Africa and other organisations to advocate for training of more pathologists and the laboratory workforce. However, the magnitude of the gaps highlighted here and by others demands that many more resources be deployed to produce higher numbers of pathologists at a faster pace in the disciplines associated with the most severe disease burdens, and to provide acceptable solutions for task shifting at each tier of the laboratory network. Key interventions to reduce the shortage of clinical pathologists include: the formulation of staffing norms in national laboratory strategic plans and healthcare human resources development strategies by defining the number and profile of pathologists at each relevant tier of the laboratory network and increasing opportunities for education, training and mentorship at the regional level, including innovative digital, remote training options. This could be done as part of the objective of the Workforce Development Institute of Africa CDC,^47^ with the establishment of certification and qualification programmes that ensure standard levels of competency, at least in national public health institutes.

### Regulatory bottlenecks slow down introduction of useful diagnostics

The past decade has seen the introduction of game-changing technologies for major public health diseases such as HIV, tuberculosis and malaria, which promise easier access, use and impact of diagnostics at the community level where most patients seek care. The WHO’s IVD prequalification process is a standardised procedure to determine whether products meet requirements for safety, quality and performance. The findings of this prequalification are used to provide guidance to countries in selecting laboratory diagnostics to be implemented at the programme level. The WHO prequalification represents the ‘ticket’ for any IVD to penetrate national markets, and is a process that takes 2–3 years. Additionally, national regulatory frameworks often foresee additional evaluations to verify that the prequalified diagnostic is adapted to specific contexts. However, the relevance of multiple, in-country evaluations is not always clear, represents unnecessary repetition, and has no additional value compared to WHO prequalification results or to a well-designed single evaluation conducted in one centre of excellence located in a region with similar disease epidemiology. Delayed registration of IVDs in-country prevents access to reliable existing diagnostics for many priority diseases including those associated with outbreaks. Regulatory bottlenecks for IVDs during country registration compromise the implementation of essential diagnostics, and prevent universal health coverage and African health security.

#### Recommendations

An innovative approach to facilitate the swift evaluation and registration of useful and performant IVD and support the advancement of the diagnostic agenda in African regions is needed. Leveraging existing networks of excellence that can quickly conduct the standardised evaluation of IVDs and support collaborative registration procedures on behalf of entire regions of Africa can lead to critical benefits in access to IVD, while waiting for or to complement WHO prequalification. The Africa Collaborative to Advance Diagnostics, led by Africa CDC, was launched in Abuja during the biannual ASLM conference in 2018^48^ with the overall aim of advocating for appropriate investment in diagnostics as well as accelerating regulations to facilitate timely and wide access to essential diagnostics. One of the goals of the Africa Collaborative to Advance Diagnostics is to work towards speedier registration of diagnostic technologies, through a pan-African approach that complements WHO prequalification, leverages existing continental expertise and provides opportunities for manufacturers and other relevant stakeholders to support the process.

### Conclusion

In addition to advancing the development of increasingly relevant, reliable, specific and affordable IVD products, the future of diagnostics in Africa also depends on our collective ability to comprehensively and swiftly address the systemic weaknesses in national laboratory networks. Under the leadership of the Africa CDC and WHO regional office for Africa, African technical agencies at the continental or regional level (e.g. ASLM, the West African Health Organization and East Central and Southern Africa Health Community) or national level (e.g. Nigeria CDC) have a critical role to play in providing direct technical assistance and vision for advancing national laboratory network diagnostic capacity and on setting adequate priorities for international cooperation. Implementing the African Union recommendations on domestic investment in healthcare^49^ is critical to ensure that laboratory systems and networks are sustainably prepared for the future of diagnostics in Africa.
